# Calcium Imaging Perspectives in Plants

**DOI:** 10.3390/ijms15033842

**Published:** 2014-03-04

**Authors:** Chidananda Nagamangala Kanchiswamy, Mickael Malnoy, Andrea Occhipinti, Massimo E. Maffei

**Affiliations:** 1 Research and Innovation Centre Genomics and Biology of Fruit Crop Department, Fondazione Edmund Mach (FEM), Istituto Agrario San Michele (IASMA), Via Mach 1, 38010 San Michele all’Adige (TN), Italy; E-Mail: mickael.malnoy@fmach.it; 2 Department of Life Sciences and Systems Biology, Innovation Centre, University of Turin, Via Quarello 15/A, 10135 Turin, Italy; E-Mails: andrea.occhipinti@unito.it (A.O.); massimo.maffei@unito.it (M.E.M.)

**Keywords:** calcium, transcription factors, calcium probes, imaging

## Abstract

The calcium ion (Ca^2+^) is a versatile intracellular messenger. It provides dynamic regulation of a vast array of gene transcriptions, protein kinases, transcription factors and other complex downstream signaling cascades. For the past six decades, intracellular Ca^2+^ concentration has been significantly studied and still many studies are under way. Our understanding of Ca^2+^ signaling and the corresponding physiological phenomenon is growing exponentially. Here we focus on the improvements made in the development of probes used for Ca^2+^ imaging and expanding the application of Ca^2+^ imaging in plant science research.

## 1. Introduction

Knowledge of Ca^2+^ signaling and its corresponding physiological phenomenon from prokaryotes to eukaryotes and from tissues to whole organisms has grown significantly. For the past six decades, calcium signaling has been a focus of study with a level of investigation higher than that of any other signaling molecule. In plants, numerous endogenous stimuli and stress signals of both biotic and abiotic nature lead to transient variation in intracellular Ca^2+^ concentration, which in turn activate respective downstream signaling cascades [1–6]. Studying the role of intracellular Ca^2+^ requires the ability to monitor the dynamics of its concentration in plant cells with both spatial and temporal accuracy [7–9].

In plants, the concentration of cytosolic Ca^2+^ ([Ca^2+^]_cyt_) is maintained in the nano molar range (100–200 nM) while in many organelles it may reach micro molar to mili molar concentrations [10,11]. Vacuole and apoplast shows mili molar Ca^2+^ concentration [12]. The use of new generation cameleon and aequorin (AEQ) has led to the discovery of Ca^2+^ dynamics at micor molar level in ER lumen and at nM level in mitochondria and peroxisomes [13–19]. In plant cells, the spatial and temporal dynamic changes of [Ca^2+^]_cyt_ induced by various stimuli determine the final functional outcome [9,20]. Therefore, in plant cells, determining changes of [Ca^2+^]_cyt_ is largely dependent on the development of methodologies that can be used to accurately measure [Ca^2+^]_cyt_ [21,22].

Here, we describe the development of various Ca^2+^ probes from the past six decades and the improvements that have been developed in this field. We also discuss the limitations of each probe and important points to consider while planning ideal Ca^2+^ imaging experiments in plant science.

## 2. Measuring [Ca^2+^]_cyt_ in Living Plant Cells

Confocal microscopy is an optical sectioning method used to reduce the image blur that is caused by inclusion of light from outside the plane-of-focus in a cross-section of a thick sample. In confocal microscopy, the path of out-of-focus light is physically blocked before detection [23]. As a comparatively non-destructive imaging technique, confocal laser scanning microscopy (CLSM) has a number of distinct advantages over alternative imaging modalities; primarily CLSM facilitates the *in situ* characterization of the 3D architecture of tissue microstructure [24]. Several papers addressed the benefits that CLSM affords during analysis of the spatial properties of intracellular [Ca^2+^] signals [20]. However, it is generally difficult to measure [Ca^2+^] in a non-invasive method and without artifacts. It is also particularly tricky to measure [Ca^2+^] in a physiologically relevant context, which allows the comparison of results obtained in the laboratory with the physiological status of plants in the field. CLSM makes extensive use of Ca^2+^ probes. However, not only CLSM can offer the possibility to couple the use of Ca^2+^ probes with *in vivo* microscopy analyses. Wide-field fluorescent microscopy has been used for both dyes and genetically encoded probes. [Ca^2+^]_cyt_ was determined using the dye Fura-2 by either fluorescence ratio with a Cairn micro photometer [25] or fluorescence ratio imaging with a GenIV-intensified Pentamax-512 charge-coupled device camera [26]. By using high-resolution deconvolution microscopy, Allen and co-workers [27] assessed the cytoplasmic localization and auto fluorescence at both emission wavelengths of cameleon, YC2.1. Recently, Costa and co-workers used Selective Plane Illumination Microscopy (SPIM) which is an imaging technique particularly suited for long term *in vivo* analysis of transparent specimens, able to visualize small organs or entire organisms, at cellular and eventually even subcellular resolution. SPIM was successfully used in calcium imaging based on Förster Resonance Energy Transfer (FRET) in *Arabidopsis* seedlings expressing the cytosolic (NES-YC3.6) or nuclear (NLS-YC3.6) localized Cameleon YC3.6 [28].

## 3. Origin and Evolution of Synthetic Ca^2+^ Indicators

In the early 1960s to 1970s, many synthetic indicators such as murexide, azo dyes, and chlortetracycline were used as Ca^2+^ indicators [29]. Among the limitations of these synthetic indicators are a low sensitivity (azo dyes), the impossibility to be used to measure Ca^2+^ in living cells (murexide) and a low accuracy (chlorotetracycline) [30]. The first fluorescent Ca^2+^ probe ideal for intracellular Ca^2+^ measurement was synthesized by Roger Tsien in the late 1980s [29]. Later on, a range of synthetic dyes was developed with desirable sensitivity, selectivity and responsiveness to measure Ca^2+^ in living plant cells [8,21]. Moreover, the evolution of AEQ [31,32], green fluorescent protein (GFP) [33] and FRET [34,35] based Ca^2+^ fluorescent imaging became more popular because of their user friendly Ca^2+^ measurement methods; however, Ca^2+^ fluorescent dyes have the advantages of being applicable to plants not suitable for transformation.

## 4. Measurement of Ca^2+^ Using Non-Ratiometric Dyes or Single Wavelength Probes

Ca^2+^ probes (also referred as sensors or reporters) are small molecules that show desirable features of sensitivity, selectivity and responsiveness to Ca^2+^. These probes form selective complexes with Ca^2+^ ions, which enable measurement of the differences in free and bound [Ca^2+^] using absorbance and emission light [36–38]. Most of the Ca^2+^ imaging probes interact with Ca^2+^ through carboxylic acid groups. This interaction causes variation in indicator properties such as fluorescent intensity and its excitation. [Ca^2+^] is not measured directly; rather the indicator monitors the amount of free and complexed probe. The concentration of free Ca^2+^ is then measured based on effective dissociation constant (*K_d_*) measured *in vitro* of the probe for Ca^2+^ in the specific environment.

[Ca^2+^]_cyt_ is measured based on relative increase in fluorescence intensity of single wavelength. Non-ratiometric dyes such as fluo, rhod and Calcium Green-1 are used for measuring [Ca^2+^]_cyt_ in plants, among them Calcium Green-11 is well-known and most commonly used in plant systems. The single excitation spectrum allows for simple instrumentation. A simple formula is used to calculate the absolute Ca^2+^ concentration in live plant cells.

$$\left[{\text{Ca}}^{2+}\right]=K_{d}\times \frac{F-F_{\text{min}}}{F_{\text{max}}-F}$$

where *K_d_* is the dissociation constant for Ca^2+^ for the fluorescent dye which is measured *in vitro; F* stands for fluorescence measured; *F*_min_ is the fluorescence of the probe in the absence of Ca^2+^ and *F*_max_ is the fluorescence of the probe at saturation point [39]. Calibration of absolute Ca^2+^ in *in vitro* samples are much easier using this method compared to ratiometric dyes but *in vivo* measurement in plant cells is a challenging task compared to animal cells. In an animal cell, it is easier to use mild detergent to permeabilize the entry of the Ca^2+^ dye through the plasma membrane, but in plants, the cell wall forms a major barrier. In plant and animal cells, limitations of this method are: corrections cannot be made for photobleaching, unequal dye loading, movements of dye within the cell and changes in optical density of the cell which affects the fluorescent intensity and accurate measurement of Ca^2+^. Moreover, these dyes are sensitive to pH, ionic strength and the surrounding protein environment, which alters their properties [7,8,21]. In order to overcome these limitations, it is recommended to simultaneously collect information on Ca^2+^-dependent fluorescence and probe concentration [30]. Other limitations such as optical artifacts could be overcome by using ratiometric dyes. These dyes offer robust approaches for *in vivo* quantitative measurement of Ca^2+^ based on fluorescence changes.

## 5. Measurement of Ca^2+^ Using Ratiometric Dyes

The excitation spectrum of ratiometric dyes Fura-2 and Indo-1, varies according to the free Ca^2+^ concentration. The Ca^2+^ concentration is measured as the ratio between two fluorescence intensity values that are taken at two wavelengths; *i.e.*, increasing wavelength λ_1_ and decreasing wavelength λ_2_. The absolute [Ca^2+^] can be calculated using the following formula:

$$\left[{\text{Ca}}^{2+}\right]=K_{d}\times \frac{R-R_{\text{min}}}{R_{\text{max}}-R}\times \frac{F_{\text{max}}^{\lambda_{1}}}{F_{\text{min}}^{\lambda_{1}}}$$

where *K_d_* is the dissociation constant, which is measured *in vitro; R* is the fluorescence ratio at both wavelengths *F*^λ2^/*F*^λ1^; *R*_min_ is the minimum ratio value (it can be at minimum or maximum calcium concentration, depending on the wavelength used in the denominator); *R*_max_ is the maximum ratio value; $F_{\text{max}}^{\lambda 1}/F_{\text{min}}^{\lambda 1}$ is a scaling factor, also known as β (*F*^λ1^ is the fluorescence used in the denominator at its maximum and minimum value).

Ratiometric dyes can be corrected for unequal dye loading, photobleaching and focal plane shift, between two cells that have the same [Ca^2+^]_cyt_. Therefore using ratios avoids many of the problems related to absolute fluorescence values. On the other hand, acquisition and data manipulation is more complex due to the use of fluorescence ratios. Not all microscopes are suitable for these measurements (changing excitation/emission wavelength at suitable rates is required) and many ratiometric indicators require the use of UV excitation, which is an expensive option for confocal laser scanning microscopy.

It is important to note that dyes are available at different ranges of *K_d_* values and spectral properties, that make them suitable for commonly used lasers [40].

## 6. Techniques of Loading Ca^2+^-Sensing Dyes into Plant Cells

Most of the Ca^2+^ interacting dyes used for Ca^2+^ measurement have highly charged groups, hence they are relatively membrane impermeable; therefore, loading probes in living plant cells is a challenging task [21]. Many loading techniques have been successfully adopted to breach the plasma membrane such as microinjection, electroporation, patch clump pipette and biolistic delivery [41–44]. Acid and ester methods are also adopted to introduce Ca^2+^ dyes; the acid method is based on pH changes. Acid loading for a 1–2 h incubation causes a drop in pH to 4.5 and protonates the dye to breach the plasma membrane in a relatively uncharged form [45,46]. Samples are incubated for 1–2 h at low pH, which might cause adverse effects on plant cells and tissues. In case of ester loading, acetoxymethyl esters are generally used for intracellular cellular cleavage by cellular esterases to deliver the Ca^2+^ dye into cytosol [30]. Ester loading causes significant hydrolysis in the plant cell wall but loading and incubation at low temperature may limit this problem [47]. Another major problem of Ca^2+^ interacting dyes is their compartmentalization in cellular organelles, mainly in the vacuole [45,48]. Compartmentalization means that the indicator is trapped within cellular organelles and not homogenously distributed throughout the plant cell [22].

It is hard to indicate which is the most efficient or the most effective dye. This mainly depends on the overlapping of emissions with autofluorescence from the tissue or the ability of the dye to penetrate the plant tissues. For example, in a recent paper, Fluo-3, which was successfully used for the calcium localization in Lima bean [49], was found to strongly interfere with the cuticular autoflourescence of *Ginkgo biloba* leaves. In this case, Calcium Orange was selected because of a different fluorescence emission [50].

Table 1 lists some of the most used Ca^2+^ dyes.

## 7. Protein Based Ca^2+^ Indicators

### 7.1. Aequorin–Based Ca^2+^ Indicators

Aequorin (AEQ) photoprotein has been extensively used in the Ca^2+^ signaling field for almost 40 years. AEQ is a Ca^2+^-binding photoprotein composed of an apoprotein (apoaequorin), which has an approximate molecular weight of 22 kDa and a prosthetic group, a luciferin molecule, coelenterazine (Mr 432). In the presence of molecular oxygen, the functional holoprotein, aequorin, reconstitutes spontaneously. The protein contains three EF-hand Ca^2+^-binding sites. When these sites are occupied by Ca^2+^, aequorin undergoes a conformational change and behaves as an oxygenase that converts coelenterazine into excited coelenteramide, which is released together with carbon dioxide. When the excited coelenteramide relax to its ground state, blue light (λ = 469 nm) is emitted [20]. This emitted light can be easily detected with a luminometer and correlates with the particular [Ca^2+^]. Identification of Ca^2+^ sensitive AEQ from *Aequorea victoria* offers enormous advantage to carry out bioluminescence research. This protein was carefully extracted and purified from jelly fish to prevent the contact with Ca^2+^ as this would cause chemiluminescence, there by rendering the protein unsuitable for Ca^2+^ measurements [61]. These proteins have been extensively engineered to obtain several luminescent probes with different biological parameters [62–64]. With the advanced genetic engineering and cloning strategies, it is possible to specifically localize them within the cell by including specific targeting amino acid sequences [41].

In the last two decades the study of Ca^2+^ dynamics in living cells has been enhanced by a significant improvement of genetically encoded protein based indicators [62]. AEQ allows their endogenous production in cell system as diverse as bacteria, yeast, fungi, plants and mammalian cells.

The new generation of bioluminescent probes coupled with Ca^2+^ sensitive AEQ allows real time measurement of Ca^2+^ changes [65,66]. Recently, AEQ based luminescent recording system has been developed to monitor spatiotemporal Ca^2+^ dynamics to various stimuli in Arabidopsis plants [63].

A major advantage of AEQ is that it can be selectively targeted to subcellular compartments by insertion of signal sequences unlike chemical compound dyes (excluding rhod-2, which is largely retained in the mitochondrial matrix). Use of recombinant AEQ facilitated the understanding of Ca^2+^ signaling interplay between different cellular compartments [67–72]. AEQ became less popular because of its small inherent signal; although the amount of signal emitted by the cell population is quite adequate to measure Ca^2+^ concentration, the signal from single cell is very low. Adequate quantity of signal is needed to overcome background noise at the expense of space and time resolutions. One of the limitations of AEQ use is that it usually overestimates the real response of cells, especially when cell suspensions are used instead of whole tissues. For instance, when comparing Fluo-3AM responses in Lima bean leaf tissues and AEQ signals in soybean cell suspensions, the upper level of estimated level of Ca^2+^ in soybean cell culture responsiveness to H_2_O_2_ corresponds to the lower level in both mechanically damaged and herbivore wounded Lima bean leaves. This situation reflects the higher sensitivity of cell suspensions cultures compared to plant tissues, and should be considered for further comparisons when AEQ is used to evaluate activities of molecules involved in signaling processes [73]. However, not only suspension cell cultures expressing AEQ have been used, but also seedlings or leaf discs. For example, Matrí and co-workers [74] used GAL4 transactivation of AEQ to analyze [Ca^2+^]_cyt_ signaling in specific cell types, including those of the leaves. Therefore, AEQ can offer tissue specificity if placed under control of tissue-specific promoters.

**Figure 1.**
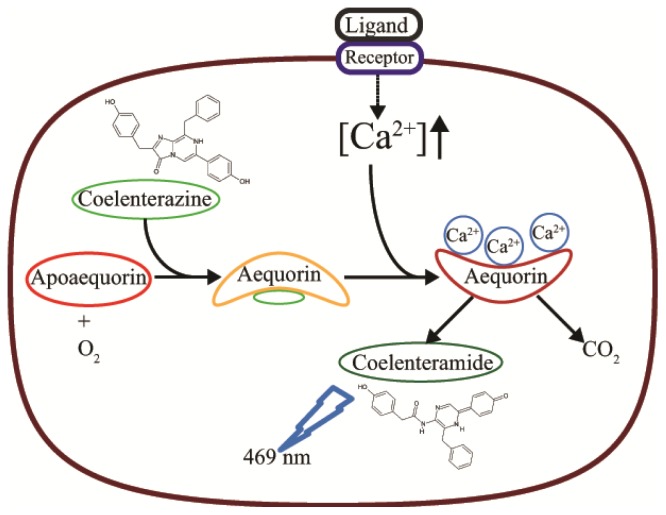
Mechanism of light emission by AEQ upon Ca^2+^-binding. The apoprotein (Apoaequorin) binds the prosthetic group Coelenterazine, a luciferine molecule. In the presence of oxygen, the holoprotein AEQ reconstitutes spontaneously. The EF-hand Ca^2+^-binding site on AEQ binds free Ca^2+^, which cause conformational changes in the aequorin. Through oxygenase activity, aequorin converts Coelenterazine into excited Coelenteramide and carbon dioxide. Coelenteramide relaxes to ground state by releasing blue light (469 nm).

In AEQ assays the emitted light is calibrated into Ca^2+^ concentrations by a method based on the calibration curve of Allen and coworkers [75]:

$$\left[{\text{Ca}}^{2+}\right]=\frac{{\left(\frac{L_{0}}{L_{\text{max}}}\right)}^{⅓}+\left[KTR\times {\left(\frac{L_{0}}{L_{\text{max}}}\right)}^{⅓}-1\right]}{KR-\left[KR\times {\left(\frac{L_{0}}{L_{\text{max}}}\right)}^{⅓}\right]}$$

where *L*_0_ is the luminescence intensity per second and *L*_max_ is the total amount of luminescence present in the entire sample over the course of the experiment. [Ca^2+^] is the calculated Ca^2+^ concentration, *KR* is the dissociation constant for the first Ca^2+^ ion to bind, and *KTR* is the binding constant of the second Ca^2+^ ion to bind to AEQ [20]. Figure 1 shows the principle of the AEQ reaction.

### 7.2. GFP–Based Ca^2+^ Indicators

GFP based Ca^2+^ sensors are immediate alternatives to synthetic dyes and AEQ described above. In 1997 the first GFP based recombinant Ca^2+^ probe was developed [33,76]. Even though there are many indicators, a limited number of those have been used in plants [77]. Currently there are three main types of this Ca^2+^ sensor: cameleons, camgaroos and pericams [61,78,79]. All these sensors were based on calmodulin (CaM) as a regulator, which changes its confirmation and alters fluorescence properties upon binding to Ca^2+^. The cameleon probe has been extensively used in plant science research as compared to camagaroos and pericams [7,8,21,27]. These probes are chimeric proteins designed on the property called change of FRET, first synthesized by Tsien and co-workers [79,80]. FRET occurs between two different colored GFP mutants, with spectral overlap of the donor emission spectrum and the acceptor absorption spectrum. In the probe, the two GFP mutants are linked together by CaM and CaM binding peptide [80]. Binding of Ca^2+^ to the Ca^2+^- responsive elements alters the efficiency of FRET. Like AEQ, several generations of FRET-based Ca^2+^ were synthesized with different biological parameters, sensitivity and efficiency; among them yellow cameleon (YC2.1) is the most popular for their use under different physiological conditions [80,81]. The YC2.1 version of cameleon has been widely used in plants for measurements of Ca^2+^ fluxes in guard cells, Nod factor responses, rhizobium and fungal colonization in the roots [27,82–85]. Later on various modified version of cameleon sensors were used to dissect subcellular Ca^2+^ dynamics; for example, D3cpv cameleon sensor for peroxisome Ca^2+^ flux and YC4.6 for pollen tube endoplasmic reticulum Ca^2+^ dynamics [17,19]. Two recent works report the use of D3cpv for mitochondrial Ca^2+^ analysis and D4ER for ER Ca^2+^ analysis [14,15]. Considering their importance in measuring Ca^2+^ dynamics at subcellular levels, efforts were made to develop a new generation of YC2.1 with significantly increased FRET signal to provide efficient Ca^2+^ measurements while reducing signal to noise ratio, this modified version was named YC3.6 [61,79,86]. This new generation with high-resolution signal has been widely used in plants for spatiotemporal imaging of cytoplasmic Ca^2+^ fluxes [87,88]. Recently YC3.6 has been successfully used to measure the cytosolic Ca^2+^ upon mechanical damage and herbivory in Arabidopsis leaves [89]. A wide range of YC sensors were successfully employed in plant science to analyze the spatiotemporal Ca^2+^ flux in different cell types such as guard cells, root, root hairs, pollen tube and different subcellular compartments [13,17,90–92]. Successes of the cameleon-based sensors are limited by CaM binding peptide as part of the sensing mechanism. Endogenous CaM could interfere with the sensor and could possibly change FRET signal.

**Figure 2.**
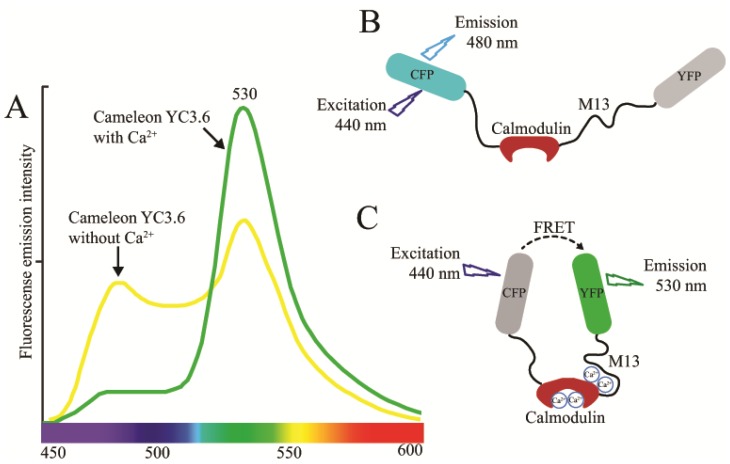
(**A**) Fluorescence emission spectrum of cameleon YC 3.6 FRET-based Ca^2+^ sensor. The increase of Ca^2+^ concentration increase the emission from YFP (FRET-acceptor); (**B**) In absence of free Ca^2+^, the donor protein (CFP) releases the absorbed energy as fluorescence at 480 nm. In the presence of Ca^2+^, the calmodulin and M13 domains bind the free Ca^2+^. The conformational change of chimeric protein allows FRET to occur between the donor fluorescent protein CFP and the acceptor fluorescent protein YFP with light emission at 530 nm.

Leaves of plants expressing FRET-based Ca^2+^ sensor YC3.6 can be ratio-imaged by CLSM. The YC3.6 Ca^2+^ sensor is usually excited at 458-nm wavelength by using an argon laser. The cyan fluorescent protein (CFP) and FRET-dependent Venus emission are assayed using a krypton/argon laser at 458 nm with a 473–505 and 526–536 nm emission filters. *In situ* calibration is performed through raising Ca^2+^ to saturating levels for YC3.6. Cells are usually permeabilized to allow a massive free diffusion of calcium inside the cell to get the *R*_max_. EGTA and EDTA can trap the free calcium released from the cells. Therefore, the maximum FRET/CFP ratio is obtained by treatment with 1 molar CaCl_2_ in response to mechanical perturbation. The minimum FRET/CFP ratio is recorded by treatment with 1 molar Tris 100 mM EDTA and 50 mM EGTA solution. [Ca^2+^]_cyt_ variations are then calculated according to the equation:

$$\left[{\text{Ca}}^{2+}\right]=\frac{K_{d}\times \left(R-R_{\text{min}}\right)}{{\left(R_{\text{max}}-R\right)}^{1/n}}$$

where *R* represents the FRET/CFP ratio measured during the experiment, *n* the Hill coefficient for YC3.6 (usually = 1), while *K_d_* values are assessed for a given concentration of Ca^2+^ [88]. Figure 2 illustrates the determination of [Ca^2+^]_cyt_ variations using a cameleon probe.

Table 2 lists some of the most used GFP-based Ca_2+_ indicators in plant sciences.

## 8. Conclusions and Future Perspectives

Imaging Ca^2+^ in living cells has seen a tremendous development in the last two decades with the evolution of genetically encoded Ca^2+^ indicators. Despite these improvements, we are still far from having an ideal Ca^2+^ probe. Here we give emphasis on future directions on the improvements of synthetic and fluorescent Ca^2+^ probes.

Currently, there are only two ratiomatric dyes fura-2 and indo-1 available since thirty years after their synthesis. Both these dyes requires UV spectrum for their excitation. As a result, there is the need for the use of expensive UV lasers for confocal microscopes, with the results of toxicity of UV illumination, lower penetration and background fluorescence. Therefore, it is necessary to develop new indicators that absorb light in the visible range.

The advent of a new generation of protein-based Ca^2+^ indicators has reduced the demand to develop new optimized dyes. Although these protein-based probes offer many advantages over synthetic dyes they are still far from being ideal probes. Most of the GFP-based probes have CaM as Ca^2+^ sensing component. CaMs are also known to be important for many physiological processes in plant system. Overexpression of GFP-based probes might results in the substantial alteration of endogenous physiological processes that depend on CaM activity [81,102]. Therefore, efforts should focus on designing new protein-based constructs as selective and sensitive as CaM but characterized by a lower or negligible interference with the endogenous components of the plant cell [102]. Further improvements on GFP-based probes should face the problem of bleaching and photoisomerization of GFP upon illumination [103]. Finding novel fluorescent protein from other organisms (other than *Aeuuorea*) would provide solutions to these problems. All these efforts will allow us to understand the complex subcellular interplay of Ca^2+^ signals underlying many plant physiological and developmental processes.

## Figures and Tables

**Table 1.** Main calcium indicators used in plant science.

Ca^2+^ indicator	Chemical structure	Properties	References
**FLUO-3**	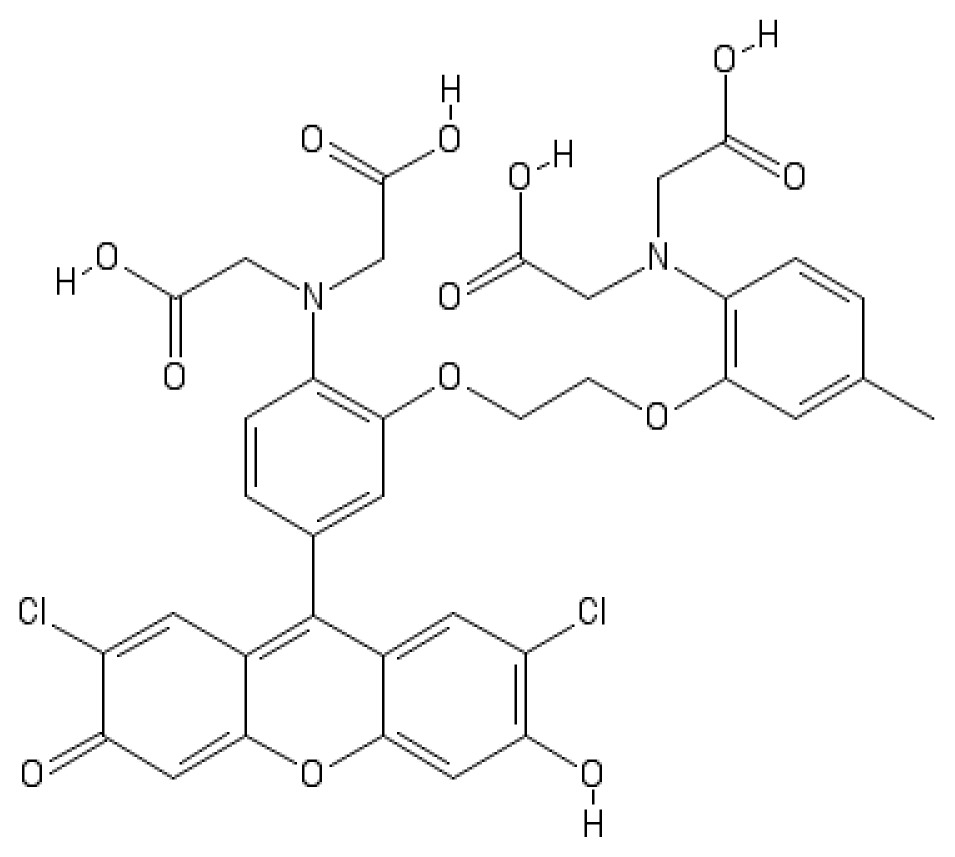	The most important properties of Fluo-3 are an absorption spectrum compatible with excitation at 488 nm by argon-ion laser sources and a very large fluorescence intensity increase in response to Ca^2+^ binding.	[49,51–53]

**FLUO-4**	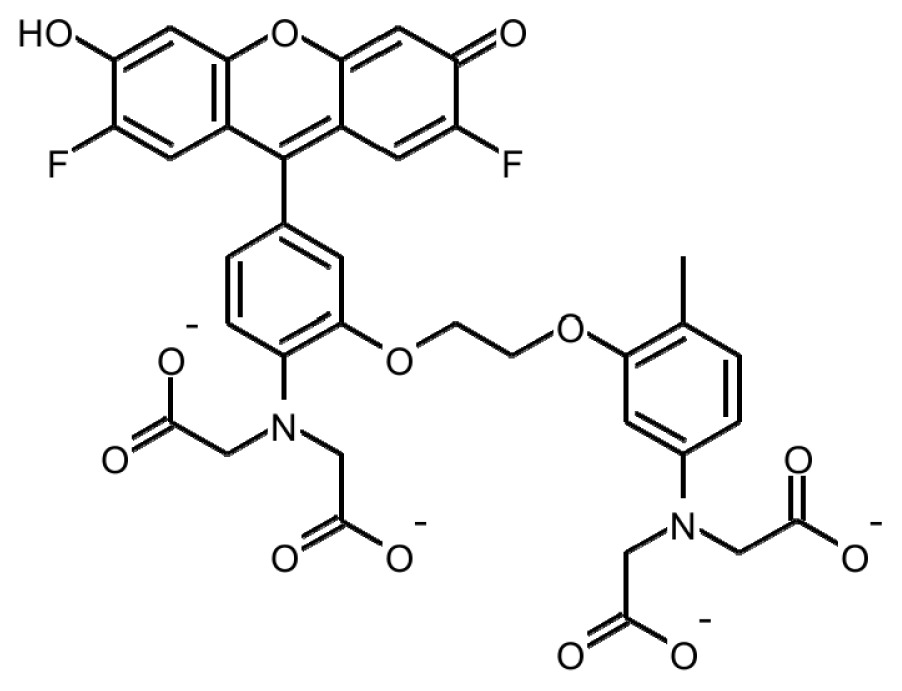	Fluo-4 and its esterified form Fluo4-AM, has a visible wavelength excitation (compatible with argon-ion laser sources) and a large fluorescence increase upon binding Ca^2+^.	[54]

**FLUO-4FF, FLUO-5F, FLUO-5N**	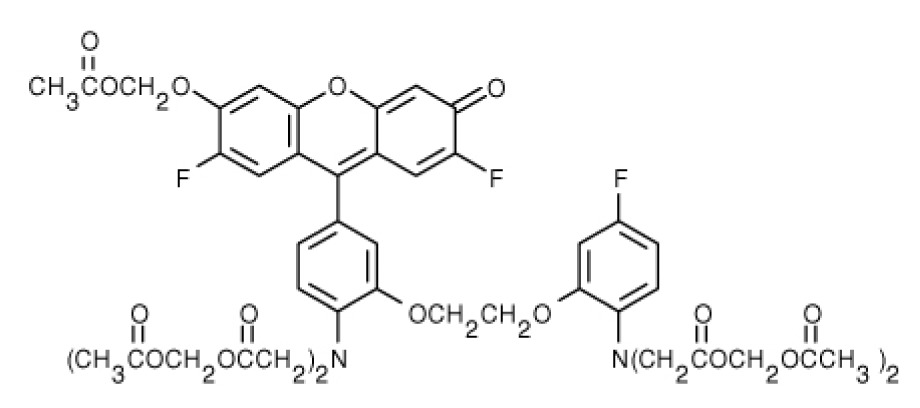	These are analogs of Fluo-4 with lower Ca^2+^- binding affinity, making them suitable for detecting intracellular calcium levels in the 1 μM to 1 mM range that would saturate the response of Fluo-3 and Fluo-4	[11]

**FLUO-4 DEXTRANS**		These are Fluo-4 coupled to a biologically inert dextran carrier (molecular weight = 10,000), providing a new and potentially valuable tool for measuring Ca^2+^ transients.	[55]

**CALCIUM GREEN™-1**	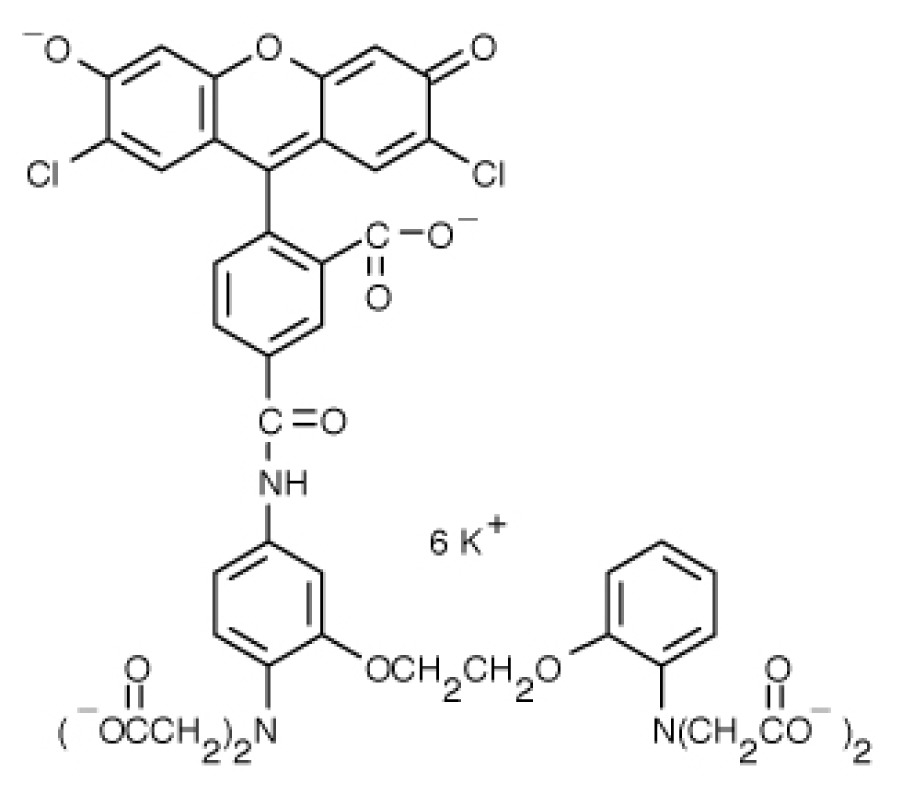	Structurally similar to Fluo-3, but is more fluorescent at low calcium concentrations, facilitating the determination of base line Ca^2+^ levels and increasing the visibility of resting cells	[56,57]

**CALCIUM GREEN™-2**	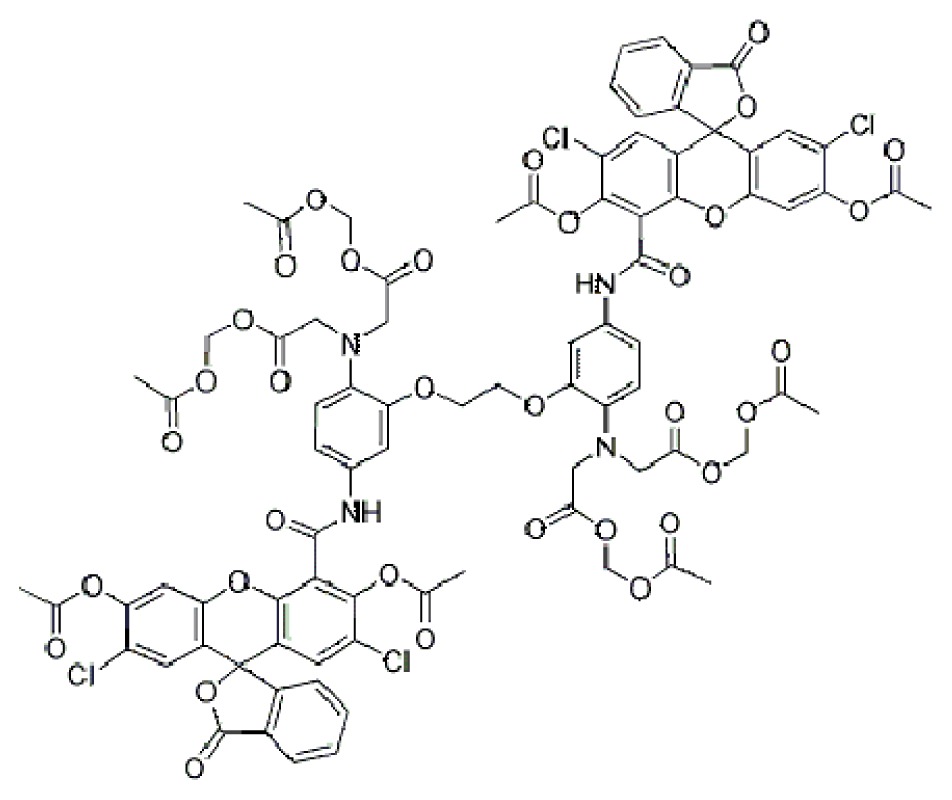	It has two fluorescent reporter groups, which are believed to quench one another in the absence of calcium, that undergo a much larger increase in fluorescence emission upon calcium binding than does Calcium Green™-1. Its lower affinity for calcium makes it particularly suited to measuring relatively high spikes of calcium, up to 25 μM.	[58]

**CALCIUM ORANGE™**	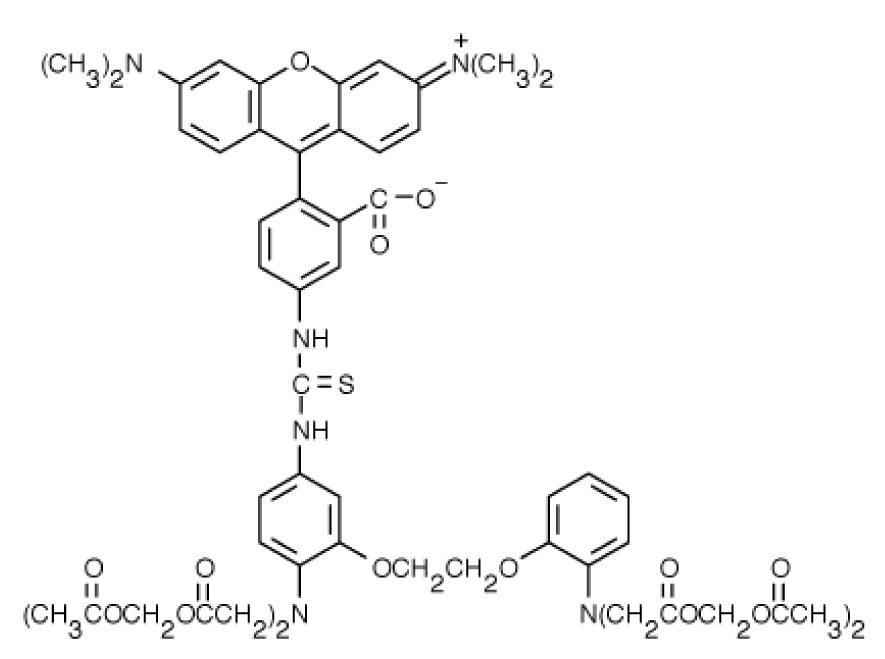	These are spectrally similar to tetramethylrhodamine and Texas Red. The long-wavelength spectral characteristics of these indicators allow them to be used in combination with fluorescein and ultraviolet excitable dyes.	[37,44,50,59]
**CALCIUM CRIMSON™**	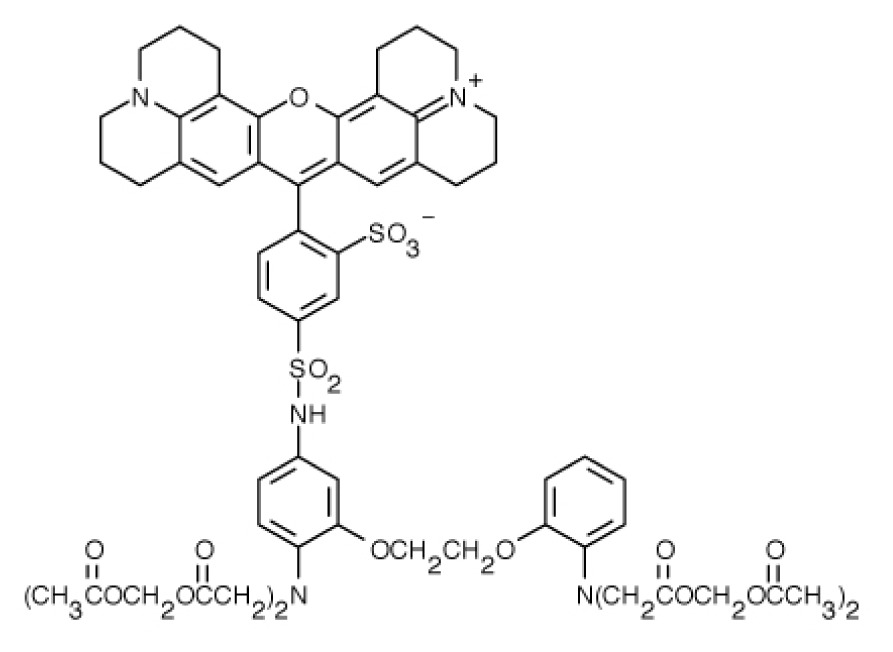		

**INDO-1**	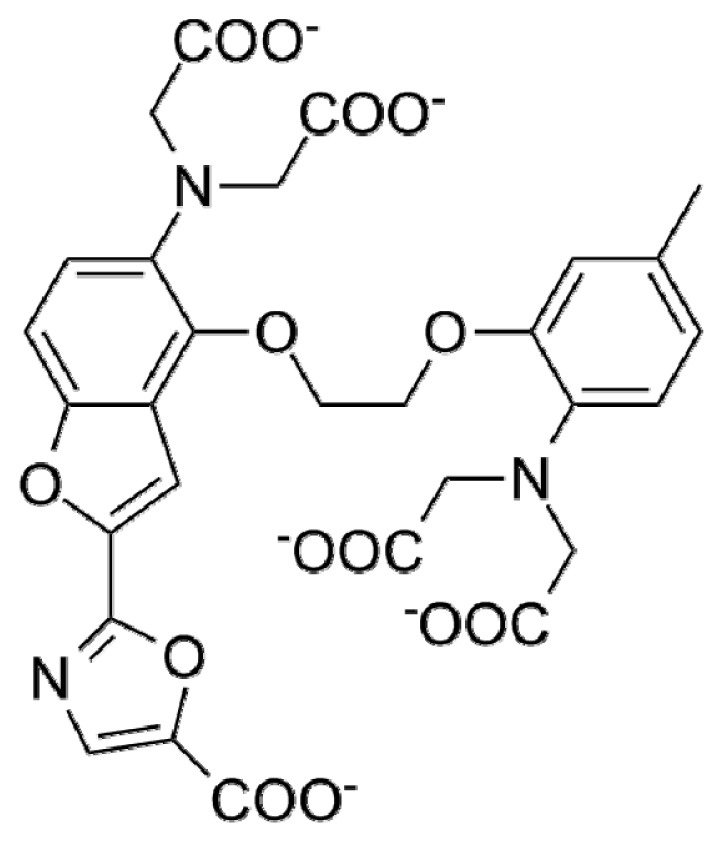	These are “dual-emission” and “dual-excitation” types of calcium dyes, respectively. To utilize either of these indicators, however, appropriate modifications of standard CLSM need to be made, such as a high-power argon-ion laser is required to obtain ultraviolet (UV) excitation, and compensatory changes along the optical path must be incorporated to deal with the lens aberrations and reduced signal throughputs that are associated with UV illuminations. At low concentrations of the indicator, use of the 340/380 nm excitation ratio for Fura-2 or the 405/485 nm emission ratio for Indo-1 allows accurate measurements of the intracellular Ca^2+^ concentration. Measurements of Indo-1 and Fura-2 fluorescence can usually be made over a period of an hour without significant loss of fluorescence resulting from either leakage or bleaching. In addition, Fura-2 and Indo-1 are bright enough to permit measurements at intracellular concentrations of dye unlikely to cause significant Ca^2+^ buffering or damping of Ca^2+^ transients.	[11,46,60]
**FURA-2**	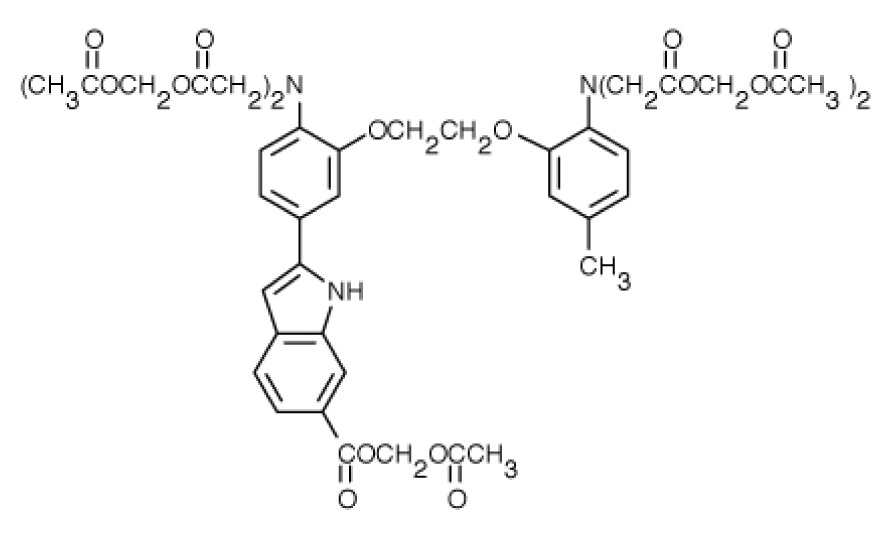		

**RHODAMINE-BASED INDICATORS.**	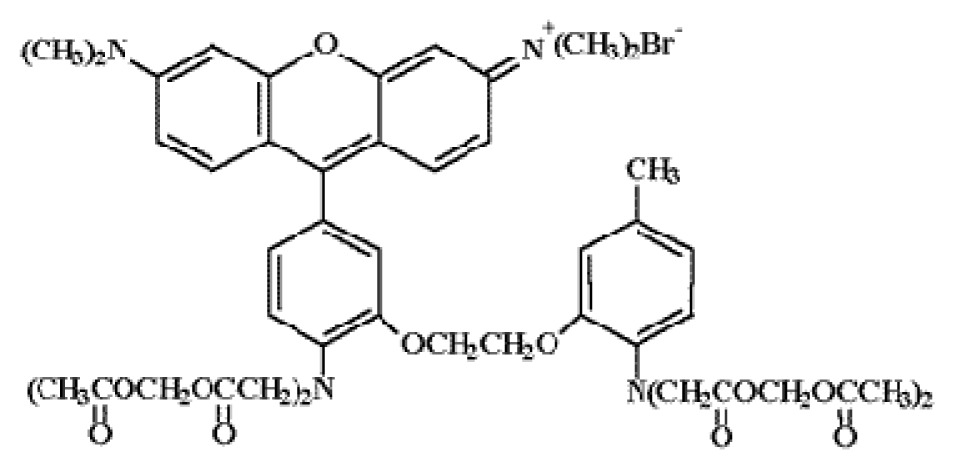	Rhod-2 has fluorescence excitation and emission maxima at 552 and 581 nm, respectively. Variants with longer-wave length excitation and emission (X-Rhod-1) and lower Ca^2+^-binding affinity (Rhod-5N, Rhod-FF, *etc*.) have been developed (*i.e.*, at Molecular Probes). Rhod-2 is used as a selective indicator for mitochondrial Ca^2+^ in most eukaryotic cells.	[11]

**Table 2.** Main GFP-based Ca^2+^ indicators used in plant science.

Cameleon family	Suitability	Stimulus/response	References
**YC2.1**	Suitable to use under various physiological condition because of their lower sensitivity to pH.	Ca^2+^ fluxes in *Arabidopsis* guard cells, Ca^2+^ elicitation in NOD factor of *medicago tranculata*, Ca^2+^ role in plant interaction with symbiotic bacteria (rhizobium) and fungal (mycorrhizal) root colonization.	[27,33,82–85,93–96]
**Nucleoplasmin- YC2.1**	Suitable for nuclear matrix localization study	Sieberer and colleague showed that Ca^2+^ spiking localized to nuclear matrix in the root hairs in response to external nodulation factors.	[97]
**D3cpv**	Suitable for studying peroxisomal Ca^2+^ dynamics	Costa and colleagues showed peroxisomal Ca^2+^ dynamics under stress signaling.	[17]
**YC4.6**	Suitable for studying endoplasmic reticulum Ca^2+^ dynamics	Iwano and colleagues showed pollen tube endoplasmic reticulum Ca^2+^ dynamics	[19]
**YC3.6**	Substitution of acceptor yellow fluorescent protein yielded five fold increased signal sensitivity, which allowed imaging of both temporal and dynamic signaling of cytosolic Ca^2+^ fluxes.	Used to study Ca^2+^ dynamics ranging from roots and root hairs, guard cells, pollen and leaves upon mechanical and herbivore damage.	[19,27,61,79,82,84, 86–89,92,94,96,98–101]
